# Mechanisms of plant virus cell-to-cell transport: new lessons from complementation studies

**DOI:** 10.3389/fpls.2024.1453464

**Published:** 2024-09-18

**Authors:** Sergey Y. Morozov, Andrey G. Solovyev

**Affiliations:** A. N. Belozersky Institute of Physico-Chemical Biology, Moscow State University, Moscow, Russia

**Keywords:** plant virus, virus cell-to-cell transport, movement protein, plasmodesmata, movement complementation, transport specificity

## Introduction

Viral movement proteins (MPs) are essential for cell-to-cell transport of plant virus genomes through plasmodesmata (PD) that connect neighboring cells in plant tissues (Lucas, 2006; Tee and Faulkner, 2024). MPs encoded by viruses of distant taxonomic groups can be unrelated in sequence, and even the number of dedicated MPs in these viruses can vary (Heinlein, 2015; Kumar and Dasgupta, 2021). Conceivably, dissimilar viral transport systems may use different mechanisms to translocate the viral genome through PD (Solovyev et al., 2022).

The *Tobacco mosaic virus* (TMV) transport system consists of a single 30-kDa MP, which was the first MP discovered and is now the best studied and considered to be the propotypic plant virus MP (Citovsky, 1999). TMV MP has several transport-related activities that enable intracellular transport of virus genome to PD and further trafficking to neighboring cells through PD. First, TMV MP binds RNA in a sequence-nonspecific manner *in vitro* and is hypothesized to interact with viral genomic RNA *in vivo* to form complexes (ribonucleoproteins, RNPs) competent for cell-to-cell transport (Citovsky, 1999). Further studies have shown that TMV MP is associated with viral replication compartments (VRCs) (Heinlein, 2015), possibly due to its RNA binding function. Second, TMV MP interacts peripherally with ER membranes and enables the transport of VRCs along the ER-actin network towards PD (Heinlein, 2015). Third, TMV MP increases the PD conductivity, or size exclusion limit (SEL), by one or more mechanisms, including (1) remodeling the internal structure of the PD channel, possibly by interacting with the PD-resident protein SYT1 (Levy et al., 2015), (2) reducing the SEL-decreasing callose depositions at the PD neck regions by activating callose-degrading enzymes (Epel, 2009), and (3) suppressing signaling that is activated in response to virus infection and leads to enhanced callose deposition (Huang et al., 2023). Intriguingly, TMV MP is also able to form tubules on the surface of MP-expressing protoplasts (Heinlein et al., 1998; Takahashi et al., 1999), reminiscent of structures typical of another class of MPs that specify a virus transport mechanism that is supposed to be fundamentally different from that of TMV MP, involving the formation of hollow tubules composed of MP subunits that replace the PD internal structure and penetrate into neighboring cells, thus serving as conduits for intercellular transport of virions (Tilsner et al., 2014). However, the functional significance of this TMV MP activity remains unknown. Importantly, the characteristic activities of TMV MP, in particular the abilities to bind RNA and to increase the PD SEL, are found in MPs of many virus transport systems unrelated to that of TMV, and can therefore be considered universal for diverse virus cell-to-cell transport mechanisms, the fine details of which remain obscure.

In recent decades, it has been well documented that cell-to-cell transport of a movement-deficient plant virus can be complemented by a heterologous, unrelated viral MP. These studies involved plant viruses of different genera, including potexviruses, hordeiviruses, tobraviruses, and carmoviruses (Atabekov et al., 1999; Latham and Wilson, 2008). Such trans-complementation of virus movement can be demonstrated using several experimental approaches, including (1) double infection with movement-competent and movement-deficient viruses; (2) inoculation of a movement-deficient virus onto transgenic plants expressing a functional MP of heterologous virus; (3) complementation *in cis* that occurs upon insertion of heterologous functional MP gene into a virus genome instead of cognate MP gene; (4) co-bombardment of plant tissues with infectious cDNA clone of a dependent virus genome and heterologous individual MP gene; (5) agroinoculation of a MP-defective virus genome construct and heterologous individual MP construct (Atabekov et al., 1999; Latham and Wilson, 2008; Zhou et al., 2019; Lazareva et al., 2022). Since at least two functions, the RNA binding and the PD SEL increase, are conserved in different MPs, complementation of viral transport can be viewed as a result of the provision of one or both of these functions by a heterologous MP. Here, we discuss recent complementation studies that may shed light on possible mechanisms of virus cell-to-cell transport complementation.

## Trans-complementation of potyvirus cell-to-cell movement by MP of plant DNA virus

Potyviruses (genus *Potyvirus*, family *Potyviridae*) have a multicomponent transport system. Potyvirus genomes encode a polyprotein precursor that is autocatalytically processed into ten mature polypeptides, several of which (CI, P3N-PIPO, HC-Pro, VPg, 6K2 and CP) are involved in viral cell-to-cell movement (Solovyev et al., 2022; Xue et al., 2023). The potyvirus cell-to-cell movement depends on the P3N-PIPO protein, which, like typical MPs, localizes to PD, increases the PD SEL, and enables its own transport through PD (Wei et al., 2010; Vijayapalani et al., 2012). P3N-PIPO interacts with CI, thereby providing a mechanism for docking of a CI-containing virus transport form, either ER-derived vesicles induced by the 6K2 protein and containing replicating virus genomes and viral proteins including CI (Xue et al., 2023), or, according to another view, modified virions with one terminus bound to HC-Pro and CI (Torrance et al., 2006; Gabrenaite-Verkhovskaya et al., 2008). Further potyviral transport through the PD channels requires the PD SEL increase, which can be induced by P3N-PIPO. P3N-PIPO is targeted to PD and directs PCaP1, a cellular Ca^2+^ cation-binding protein that normally interacts with the PM via myristoylation, to PD, where the Ca2+-dependent actin filament-severing activity of PCaP1 enables the SEL increase either by affecting local callose deposition (Cheng et al., 2020) and/or by disrupting actin filaments in the PD channels (Schreiber et al., 2024). Thus, P3N-PIPO is the key component of the potyviral viral transport machinery operating at PD.

A single amino acid substitution (K15E) in P3N-PIPO results in defective virus cell-to-cell movement (Gong et al., 2022). Strikingly, this deficiency can be partially complemented by a plant DNA virus MP coexpressed *in trans*. This relatively small MP (67 aa in length), designated C5, is encoded by the genome of monopartite begomovirus *Tomato yellow leaf curl virus* (family *Geminiviridae*) (Zhao et al., 2023). The GFP-fused C5 protein localizes to PD and can move between cells through PD (Zhao et al., 2023). The C5 protein can target V2, another virus protein, which is associated with the ER and localized to the nucleus when expressed alone, to PD. Since V2 interacts with the viral capsid protein, one of the functions of V2 has been proposed to be an adaptor protein in the C5-mediated targeting of virions to PD (Zhao et al., 2023). Similar to the begomovirus C5 protein, a P3 protein encoded by *Diaporthe sojae circular DNA virus 1*, an ssDNA fungal virus that infects plants, localizes to PD in plant tissues and can complement the cell-to-cell movement of a P3N-PIPO-deficient potyvirus (Wang et al., 2024). It should be noted, however, that the geminivirus C5 and the putative MP of the fungal virus have no significant sequence similarity.

The ability of the two proteins, geminivirus C5 and P3 of the fungal virus, both encoded by DNA viruses, to complement RNA virus transport cannot be explained by their possible ability to bind potyvirus genomic RNA and thus form a transport-competent entity. First, the potyvirus transport form is not MP-coated RNA, but rather modified virions or membrane vesicles (see above). Second, it is very unlikely that DNA virus-specific MPs, which are dedicated to cell-to-cell transport of DNA genomes, have RNA binding activity. On the other hand, both the C5 and P3 proteins complement the functions of P3N-PIPO, which is known to be localized to PD and function at PD. Therefore, the complementation of P3N-PIPO functions by the C5 and P3 proteins, which are both found in PD, can be explained by the PD modification by these proteins, likely as a result of interaction with PCaP1, remorin, or other PD-resident polypeptides, leading to actin remodeling and/or inhibition of callose deposition. Therefore, we assume that the complementation of heterologous virus movement by the C5 and P3 proteins can be based solely on their ability to modify PD.

## Trans-complementation of viroid cell-to-cell movement by plant virus MP

Viroids are small infectious circular ssRNAs that infect a wide range of plants (Ortolá and Daròs, 2023; Zhang et al., 2024). Since viroids are non-coding RNAs, their cell-to-cell and long-distance movement depends on cell proteins and specific signals in viroid RNA that can be recognized by these proteins (Tolstyko et al., 2020). In a similar manner, trafficking of two phloem-limited umbravirus-like viruses (ULVs) that do not encode MPs involves phloem proteins such as PP2, which binds ULV RNA (Ying et al., 2024). The circular RNA genome of *Potato spindle tuber viroid* (PSTVd) folds into a rigid secondary structure composed of stem-loops (Ding, 2009). These secondary structure elements involve non-canonical base pairs and other non-Watson-Crick nucleotide interactions that form structurally unique three-dimensional motifs that are specifically recognized by cell proteins involved in PSTVd replication and trafficking (Tolstyko et al., 2020). Indeed, mutations in several structural elements of the viroid RNA are found to inhibit its transport between plant cells (Ma et al., 2023; Zhang et al., 2024). In particular, a mutation in loop 27 of the PSTVd RNA blocks viroid transport from epidermal to palisade mesophyll cells (Takeda et al., 2011). Strikingly, the transport of the mutant between these cell types can be complemented by the *Tobacco mosaic virus* (TMV) MP (Wu and Bisaro, 2022). At first glance, this observation might indicate the functional replacement of a plant RNA-binding protein that specifically interacts with the PSTVd loop 27 by the viral MP. However, this interpretation is difficult to correlate with the fact that TMV MP does not selectively bind PSTVd RNA compared to ssRNA and DNA (Wu and Bisaro, 2022). On the other hand, since the TMV MP increases the SEL of PD between mesophyll cells in mature tobacco leaves (Wolf et al., 1989; Deom et al., 1990), it can be proposed that PD modification is the primary mechanism underlying the ability of TMV MP to complement viroid movement. Consistent with this suggestion, the earlier observations that TMV MP can increase the SEL of PD between mesophyll and bundle sheath cells, but not between bundle sheath and phloem parenchyma cells (Ding et al., 1992), correlate with the inability of TMV MP to mediate phloem entry of the 76AU and 156AU PSTVd mutants (Wu and Bisaro, 2022). Thus, the available data suggest that TMV MP complements PSTVd transport by modulating the conductivity of the PD channels.

## Specificity of transport through PD

In the examples discussed above, the viral MPs capable of complementing cell-to-cell transport of heterologous virus or even viroid genomes appear to act in a non-specific manner, i.e., without any specific interactions with proteins of the complemented virus or virus/viroid RNA, most likely solely by PD modification and the PD SEL increase. Since viral cell-to-cell transport is considered to be a more complex process than a simple genome “snaking” through the open PD gate, it remains unclear how a change in the PD conductivity can be sufficient for transport complementation. Therefore, the studies discussed in this paper reopen the long-debated question of how viral MPs provide selective intercellular transport for cognate viral RNA genomes. Selectivity may depend on specific binding of viral RNA by MPs; however, where experimental data are available, MPs have been shown to bind RNA non-specifically. Another apparent way to provide the transport specificity is coupling of viral RNA replication with virus transport. In this model, viral replication compartments are located in close vicinity of PD, and nascent RNA progeny is forwarded by MPs directly to the PD channels (Tilsner et al., 2013). In addition, a certain level of selectivity may be provided by specific interaction of MPs with viral replicative proteins leading to the targeting of replicative compartments to PD (Wu et al., 2019). These mechanisms imply the incorporation of MPs into viral replicative compartments that is often observed in natural infection and is not the case in the complementation studies discussed above, especially in the movement complementation of viroid, which does not form replication compartments at the PD entrance and is replicated in the nucleus by cell proteins (Zhang et al., 2024). Thus, the above discussed examples of viral movement complementation between distant RNA and DNA infectious agents prompt researchers to identify the mechanism of specificity of MP functions in virus cell-to-cell transport.
